# Detection and Quantification of Ammonia as the Ammonium Cation in Human Saliva by ^1^H NMR: A Promising Probe for Health Status Monitoring, with Special Reference to Cancer

**DOI:** 10.3390/metabo13070792

**Published:** 2023-06-26

**Authors:** Mohammed Bhogadia, Mark Edgar, Kayleigh Hunwin, Georgina Page, Martin Grootveld

**Affiliations:** Leicester School of Pharmacy, De Montfort University, The Gateway, Leicester LE1 9BH, UK; mohammed.bhogadia@dmu.ac.uk (M.B.); mark.edgar@dmu.ac.uk (M.E.); p2421485@my365.dmu.ac.uk (K.H.); p16198195@alumni365.dmu.ac.uk (G.P.)

**Keywords:** ammonia/ammonium ion, human saliva, ^1^H NMR analysis, NMR-based metabolomics, metabolites, exchangeable ^1^H nuclei, calibration, signal-to-noise ratio, hyper-ammonaemia, chronic kidney disease, cancers

## Abstract

Ammonia (NH_3_) has been shown to be a key biomarker for a wide variety of diseases, such as hepatic and chronic kidney diseases (CKD), and cancers. It also has relevance to the oral health research area, and, hence, its determination in appropriate biofluids and tissues is of much importance. However, since it contains exchangeable >N-H protons, its analysis via ^1^H NMR spectroscopy, which is a widely employed technique in untargeted metabolomic studies, is rendered complicated. In this study, we focused on the ^1^H NMR analysis of this biomarker in less invasively collected human saliva samples, and we successfully identified and quantified it as ammonium cation (NH_4_^+^) in post-collection acidulated forms of this biofluid using both the standard calibration curve and standard addition method (SAM) approaches. For this purpose, *n* = 27 whole mouth saliva (WMS) samples were provided by healthy human participants, and all donors were required to follow a fasting/oral environment abstention period of 8 h prior to collection. Following acidification (pH 2.00), diluted WMS supernatant samples treated with 10% (*v*/*v*) D_2_O underwent ^1^H NMR analysis (600 MHz). The acquired results demonstrated that NH_4_^+^ can be reliably determined in these supernatants via integration of the central line of its characteristic 1:1:1 intensity triplet resonance (complete spectral range δ = 6.97–7.21 ppm). Experiments performed also demonstrated that any urease-catalysed NH_3_ generation occurring post-sampling in WMS samples did not affect the results acquired during the usual timespan of laboratory processing required prior to analysis. Further experiments demonstrated that oral mouth-rinsing episodes conducted prior to sample collection, as reported in previous studies, gave rise to major decreases in salivary NH_4_^+^ levels thereafter, which renormalised to only 50–60% of their basal control concentrations at the 180-min post-rinsing time point. Therefore, the WMS sample collection method employed significantly affected the absolute levels of this analyte. The LLOD was 60 μmol/L with 128 scans. The mean ± SD salivary NH_4_^+^ concentration of WMS supernatants was 11.4 ± 4.5 mmol/L. The potential extension of these analytical strategies to the screening of other metabolites with exchangeable ^1^H nuclei is discussed, as is their relevance to the monitoring of human disorders involving the excessive generation and/or uptake of cellular/tissue material, or altered homeostasis, in NH_3_.

## 1. Introduction

The production of ammonia (NH_3_) is a highly ubiquitous process within the human body, where it is generated from different precursor molecules, such as arginine via arginine deiminase or urea through urease [1], with these enzymes being widely expressed in host-sourced bacteria, fungi and algae [2]. In view of its known toxicological effects, concentrations of NH_3_ in vivo are required to be tightly regulated, since elevated levels can lead to hyper-ammonaemia, which, in turn, can give rise to severe neurological complications [3]. Such adverse effects emphasise the importance of maintaining the homeostasis of this toxin. Several studies showed that NH_3_ levels in human plasma are upregulated in patients with liver disease, which is caused by disturbances in the hepatic conversion of NH_3_ to urea via the well-known urea cycle [4,5,6]. Furthermore, Kopstein and Wrong [7] showed that plasma levels of the NH_3_/ammonium ion (NH_4_^+^) precursor urea are correlated with those of its salivary counterpart, and it is now generally accepted that human saliva may also be employed in the determination of key biomarker metabolites, which have the capacity to identify and diagnose a wide range of human diseases, including remote systemic and oral disorders [8,9,10]. These developments offer many advantages, since this biofluid is significantly easier to collect, with the procedure usually being much less invasive than the collection of whole blood, usually for plasma or serum analysis. Therefore, this approach may also serve to provide useful information on diseases associated with, or indicated by, excessive concentrations of NH_3_ [7].

Notably, levels of salivary NH_3_ were shown to be upregulated in dental caries-free healthy human participants relative to those who have this oral health condition, and these differences result from increases in the activities of the deiminase and bacterial urease enzyme systems [11]. Given the fact that NH_3_ is a proton acceptor (i.e., a weak Lewis base), the higher pH, acid-neutralising conditions generated create an unfavourable environment for the growth and preponderance of cariogenic micro-organisms. Moreover, salivary urea levels are also shown to be upregulated in patients with chronic kidney disease (CKD), with a common symptom being ‘ammonia breath’ caused by excess urea not being removed by the kidneys, which then enters saliva and is degraded to ammonia by bacterial urease enzymes [12]. Therefore, these studies highlight the importance of deriving a suitable convenient bioanalytical protocol and methodology to accurately detect and quantify ammonia and/or its protonated form (NH_4_^+^ ion) in human saliva as a potential diagnostics tool for selected diseases.

As reviewed in Ref. [13], previously reported mean healthy reference human NH_3_ concentrations in a range of biofluids/biosamples were 12–98 and 21–57 μmol/L in venous blood and venous plasma, respectively; 26–35 mmol/L in 24-h urine samples; 9–20 μmol/L in cerebrospinal fluid (CSF); 110 μmol/L in bile; 1.9–3.0 mmol/L in sweat; 11.2 mmol/L in faeces; and 4.4 mmol/L in saliva.

Recently, a series of investigations featured determinations of NH_3_ in the human oral environment using ‘state-of-the-art’ biosensor technologies. Indeed, such approaches have largely involved its analysis in human breath, and examples of previous studies that used this gaseous analyte medium have involved its detection and quantification as a biomarker for a wide range of human diseases, including asthma, lung metabolism of monoamines, kidney insufficiencies (including toxic kidney damage, hypertonic disease, nephritises, and toxicosis) liver insufficiency, hepatitis, cirrhosis and toxic hepatitis, acute and chronic radiation exposure diseases, and, most notably, lung cancer [14,15]. Its mean healthy human breath concentration was estimated to be 265 ppb (range 29–688 ppb) [16], which is equivalent to a level of only 16 μmol/L. Intriguingly, a comparison between oral and nasal breath in a small number of participants revealed that NH_3_ concentrations in the latter group were significantly lower than corresponding oral concentrations [16].

Further human breath-based studies involved an exploration of the ability of ureolytic bacteria present in supragingival plaque to metabolise urea to NH_3_ and carbonic acid/carbonate for the screening of CKD [17], an investigation into biochemical routes available for NH_3_ production in end-stage renal disease patients who underwent haemodialysis [18] (this study also demonstrated quite powerful correlations between blood and salivary urea levels, and that the latter factor was also correlated to salivary NH_3_ concentrations), and a study of the correlation between human breath NH_3_ and blood urea nitrogen (BUN) concentrations in both CKD and dialysis patients [19], as well as its use as an oral biomarker for the prediction of oral malodour [20].

Of particular interest is the knowledge that NH_3_, along with amine vapours, such as hydrazine and triethylamine, which are highly toxic to humans, can serve as biomarkers for bronchogenic and prostate cancers [21]. Notably, Das et al. [22] suggested that a hydrogel/phenazine-based smartphone-assisted ‘optic electronic nose’ was a very useful tool for the selective recognition of NH_3_ and related amine compounds for early-stage cancer diagnosis. Indeed, breath testing serves as an invaluable low-cost non-invasive strategy that may permit the diagnosis of lung, and, potentially, a range of other, cancers. Also, Changsen et al. [23] found that urea, as a by-product of NH_3_ metabolism, could serve as a potential serum biomarker for hepatocellular carcinoma (HCC).

Recently, Bell et al. [24] found that microenvironmental NH_3_ facilitates T cell exhaustion in colorectal cancer. Of particular interest, clearance of NH_3_ was found to reactivate these T cells and, overall, decrease the development and extent of colorectal cancer in these experimental animals.

In 2021, Navaneethan et al. [25] explored patterns of volatile organic compounds (VOCs) in bile in order to distinguish patients with pancreatic cancer from those with chronic pancreatitis. They found that a combination of NH_3_, acetonitrile, and trimethylamine successfully detected pancreatic cancer patients with a sensitivity and specificity of 93.5 and 100%, respectively, and concluded that the determination of these VOCs in bile allowed the accurate distinction of these disorders.

In principle, the urea cycle may serve to effectively remove high and, hence, toxic concentrations of NH_3_/NH_4_^+^, which may, indeed, facilitate cancer cell proliferation. Therefore, as a by-product of NH_3_ metabolism, urea may act as a valuable biomarker for selected carcinomas, for example, HCC. Moreover, such applications may also extend to the salivary monitoring of both urea and its metabolic precursor, NH_3_, for this diagnostic purpose.

Biomarker quantification via solution-based NMR techniques offers several advantages over frequently used liquid chromatography-mass spectrometry (LC-MS) approaches, one of which is its ability to virtually retain the ‘natural’ state of the biofluid in question. This function is particularly important and of much significance, since the technique has the ability to virtually retain the physiological environment of biofluids investigated, and, hence, ‘preserves’ the physicochemical properties of monitored metabolites within a ‘natural’, untampered environment. These advantages also allow considerations and studies of the interactions and solution-state equilibria of low-molecular-mass metabolites with biofluid macromolecules, which are predominantly proteins, while conducting standard metabolomics investigations. It also has the capacity to provide information and report on the response of low-molecular-mass metabolites to changes in biofluid pH, ionic strength, and complexation equilibria with metal ions, particularly those of calcium (Ca^2+^) and magnesium (Mg^2+^) [26], amongst others. Hence, biomedical ^1^H NMR analysis offers many bioanalytical benefits, being significantly more information-rich than alternative techniques, such as the sample-destructive LC-MS strategies, which is an advantage that renders it more suitable for deployment in high-level untargeted metabolomics studies.

To date, several previous studies have employed ^1^H NMR analysis to quantify levels of NH_4_^+^ ion, both in biological and environmental samples, along with necessary authentic pure calibration standards (usually containing ammonium chloride), and each of the methods utilised highlighted limitations regarding its accurate quantification in view of solvent-exchangeable protons present in the NH_4_^+^ analyte. Indeed, these protons readily exchange with relatively lower levels of ^2^H_2_O present in aqueous solvent media that is predominantly used for analyses such as a field frequency lock, in addition to a marked suppression in intensity caused by now commonly used pre-saturation pulse sequences [5,10,27,28]. For biofluids, however, to date, these investigation(s) have been limited to NH_4_^+^ ion detection in blood plasma [5,29]. Furthermore, most metabolomics studies are almost exclusively conducted at physiological or near-physiological pH values, at which point the ^1^H NMR resonances of NH_4_^+^ would be ^1^H NMR-invisible; indeed, they only become visible at pH values < 3.5 [5].

In view of the potential role of NH_3_ as a significant diagnostic biomarker, in this study, we employed the ^1^H NMR technique at a high operating frequency (600 MHz) to determine its concentrations in clear supernatants derived from whole human saliva (WMS) samples collected from a range of healthy human participants. For this purpose, we employed a range of different experimental approaches in order to circumvent some of the above issues and limitations. To the best of our knowledge, this paper is the very first full report available on the ^1^H NMR-based determination of NH_3_/NH_4_^+^ ion in human saliva. Such developments are of much importance, since appropriate methodologies for the identification and quantification of NH_3_ and its corresponding charged cation in biofluid samples will potentially offer significant benefits in terms of the screening of human diseases, including cancers. Extensions of the bioanalytical strategies used for the determination of alternative, non-ammoniacal metabolites with exchangeable protons are also discussed.

## 2. Materials and Methods

### 2.1. Saliva Sample Collection and Preliminary Laboratory Processing

Multiple WMS samples were collected from a total of *n* = 7 healthy human participants (3 male/4 female), with a mean ± SEM age of 34 ± 5.67 years (range 21–59 years), who provided these samples on different sampling days after a minimum duration of 8 h of oral environment abstention/fasting episodes and using the passive drool approach, as previously reported in [8], which expectorated as much WMS sample as possible during a 1–2-min period. A total of *n* = 27 WMS samples were used for our ^1^H NMR experiments. Samples were immediately centrifuged at 11,000 rpm for a period of 10 min at 4 °C in order to remove cells and debris, and the clear supernatants (WMSSs) were then stored at −80 °C until becoming ready for ^1^H NMR analysis.

All studies were conducted according to the guidelines of the Declaration of Helsinki. The investigations described were approved by the Research Ethics Committee of the Faculty of Health and Life Sciences, De Montfort University (DMU), Leicester, UK (reference nos. 1082 and 457,249). Written informed consent was obtained from all participants involved in the outlined studies. No participants recruited to the study provided any identifying information: all samples and their ^1^H NMR analysis records were completely anonymised according to our ethics committee requirements.

### 2.2. Preparation of WMSS Samples for ^1^H NMR Analysis

For the ^1^H NMR determination of salivary NH_4_^+^ ion concentrations, 0.10 mL aliquots of WMSS samples were diluted to a final volume of 0.70 mL of an aqueous medium containing dilute hydrochloric acid (HCl) and a chemical shift reference and internal quantitative ^1^H NMR standard sodium 3-(trimethylsilyl)propionate-2,2,3,3-d_4_ (TSP, Sigma-Aldrich Chemical Co., Ltd., Gillingham, UK) of purity 98 atom %deuterated TSP, as well as 10% (*v*/*v*) D_2_O (Fisher Scientific, Loughborough, UK). The final HCl and TSP concentrations of these analyte solutions were 20 mmol/L and 250 μmol/L, respectively, and the final pH value was 2 (range 1.90–2.10). The pH values of each final analyte solution were predominantly found within this range; in a few samples that had slightly higher pH values, very small μL volumes of 1 mol/L HCl were added to reduce pH to within this threshold range, and the final analyte (NH_4_^+^) concentration determined was marginally adjusted to account for this further, albeit very minor, dilution. The pH values of analytical samples were determined using a hand-held Accument AET 15 pH meter (Fisher Scientific Ltd., Loughborough, UK).

The concentration of NH_4_^+^ ion in the WMSS samples was then determined using the standard calibration curve method (Section 2.5); these levels were computed after accounting for the above-mentioned sample dilution protocol(s).

### 2.3. Acquisition of ^1^H NMR Spectra

All samples were acquired via a Jeol JNM-ECZ600R/S1 spectrometer operating at a frequency of 600.173 MHz. ^1^H NMR acquisition parameters were as follows: 128 or 1024 scans (unless otherwise stated), a 1-s relaxation delay, a 1.82-s acquisition time for each free induction decay (FID), 16,384 data points, 8.99 kHz as a sweep-width, and an operating temperature of 298 K. The WET-2 [30] or ROBUST-5 [31] pulse sequences [32] were employed for the acquisition of spectra, with the irradiation frequency set at δ = 4.68 ppm, a pulse power of 8.3 dB, and a pulse duration of 7.5 µs. For finalised NH_4_^+^ determinations using our newly-developed, recommended methods, the ROBUST-5 pulse sequence was employed.

### 2.4. Investigations of the Influence of Increasing Solution D_2_O Contents on ^1^H NMR Spectral Profiles

Authentic samples of ammonium chloride (5 mM) were prepared in a final volume of 0.60 mL containing 20 mM HCl (pH 2), with 5, 10, 50, and 90% (*v*/*v*) contents of D_2_O added to the sample analyte solutions. Samples were placed into 5-millimetre diameter NMR tubes (Norrell, NC, USA) and then acquired via the WET pulse sequence using the parameters provided in Section 2.3

### 2.5. Generation of Calibration Curves

**Figure 1:**
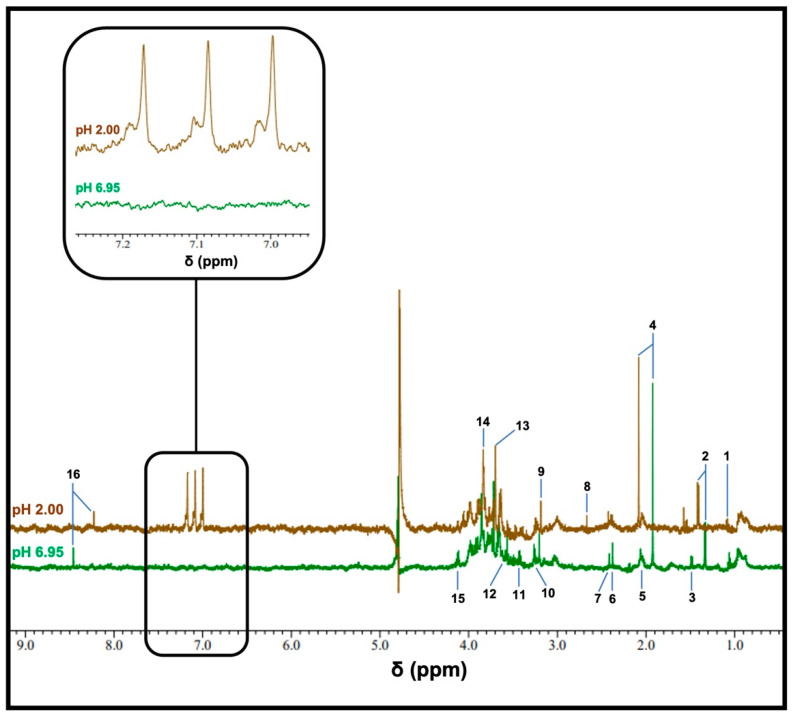
The typical ^1^H NMR spectra of a WMSS sample in a 90% H_2_O/10% D_2_O (*v*/*v*) solvent system. Spectra of WMSSs (following a 1/7 (*v*/*v*) dilution) were acquired at pH 6.95 (green), and following the addition of 20 mM HCl (final concentration), yielded a final pH value of 2 (brown). These profiles were obtained with 128 scans using the ROBUST-5 pulse sequence. The expanded region shows the 6.95–7.26 ppm chemical shift range of the pH 2 spectrum, which contains the clearly-visible NH_4_^+^ ion triplet resonance. Assignment abbreviations: 1, propionate-CH_3_ (*t*); 2, lactate-CH_3_ (*d*); 3, alanine-CH_3_ (*d*); 3, acetate-CH_3_ (*s*); 5, low- and high-molecular-mass N-acetylsugar-NHCOCH_3_, with the latter being ascribable to the molecularly mobile carbohydrate side-chains of ‘acute-phase’ glycoproteins (sharp and broad singlets, respectively); 6, pyruvate-CH_3_ (*s*); 7, succinate-CH_2_s (s); 8, unassigned signal; 9, choline-N(CH_3_)_3_^+^ (*s*); 10 and 11, taurine-CH_2_NH_3_^+^ (*t*) and -CH_2_SO_3_^-^ (*t*) resonances, respectively; 12, glycine-CH_2_ (*s*); 13, guanidoacetate-CH_2_ (); 14, glycolate-CH_2_OH (*s*); 15, lactate-CH (*q*); 16, formate-H (*s*).

Ammonium chloride standard solutions of concentrations in the range 0.25–20.0 mmol/L were prepared individually in triplicate in a final volume of 0.70 mL, with each sample containing 20 mmol/L HCl and 250 μmol/L TSP, along with 10% (*v*/*v*) D_2_O. Samples were placed in 5-millmetre diameter NMR tubes, and ^1^H NMR spectra were acquired using the ROBUST-5 pulse sequence. The central NH_4_^+^ ion line of its signal (Figure 1) was electronically integrated and expressed relative to that of the TSP signal located at δ = 0 ppm. This approach was selected in view of potential superimpositional interferences to the higher- (δ = 6.99) and lower-field (δ = 7.17 ppm) lines of the NH_4_^+^ ion 1:1:1 intensity triplet, which arose from the ^1^H NMR resonances of other endogenous salivary metabolites, e.g., tyrosine and further selected phenolic biomolecules.

Normalised integrals were plotted as functions of the NH_4_^+^ ion concentration, and data points were fitted to a linear regression model using GraphPad prism version 9.0. The final concentration of this target analyte in WMSS samples was determined using the standard calibration curve.

### 2.6. Standard Addition Method (SAM) for Salivary NH_4_^+^ ion Determinations: Sample Preparation

Pre-fixed volumes (0.50 mL) of human WMSSs were removed and diluted to a final volume of 0.70 mL, with each analyte solution containing final levels of 20 mM HCl and 250 μmol/L TSP, as well as added ammonium chloride (Fisher Scientific Ltd., Loughborough, UK) standard solutions containing NH_4_^+^ concentrations in the range 7–216 mmol/L and 10% D_2_O (*v*/*v*). Separate replicate samples were prepared for each added NH_4_^+^ ion concentration and placed into 5-mm NMR tubes. ^1^H NMR spectra were acquired using the ROBUST-5 pulse sequence [31]. The central line of the NH_4_^+^ ion triplet signal (δ = 7.08–7.14 ppm) was integrated and expressed relative to that of the added TSP internal standard resonance at δ = 0 ppm. Normalised integrals were plotted as a function of the added ammonium chloride concentration, and datapoints were fitted to a linear regression model using GraphPad prism version 9.0. The salivary concentrations of NH_4_^+^ ion were determined based on the generated equation and the negative abscissa intercept value.

### 2.7. Time-Dependent Determinations of Salivary Ammonia/Ammonium Ion Concentration following WMS Sample Collection and Laboratory Processing

Immediately following collection, fresh pre-fasted high volume independent WMS samples (*n* = 3) were centrifuged at 11,000 rpm for a period of 10 min at 4 °C. The clear WMSSs were then removed, and from these WMSSs, an initial sample volume of 0.10 mL was transferred to 5-millmetre diameter NMR tubes for ^1^H NMR analysis in solutions containing final concentrations of 2 mmol/L HCl and 250 μmol/L TSP, as well as 10% (*v*/*v*) D_2_O (final volume 0.70 mL). The remainders of each WMSS sample were then equilibrated at 37 °C in a water bath, and, subsequently, 0.10 mL aliquots were removed at 60-min interval time points for up to 6 h prior to acidification with HCl for ^1^H NMR analysis, as noted above. A final sample was collected at the 18-h equilibration time point and similarly prepared and analysed. ^1^H NMR spectra were again acquired with the ROBUST-5 pulse sequence using the parameters described in Section 2.3.

Additional experiments were focused on the potential interference of NH_3_ generation via salivary urease post-sample collection, as well as on methods for the circumvention of this process. Immediately following collection, *n* = 3 separate fresh WMS samples from a single 8-h pre-fasted participant that were collected on three different days were centrifuged at 11,000 rpm. for a 10-min period at 4 °C. The clear WMSSs were then removed, and samples were either treated with 5 mmol/L sodium fluoride, which is a known urease inhibitor [33] (Fisher, UK), or, alternatively, filtered using 3 kDa cut-off Amicon microfiltration devices (Millipore, UK), and from these samples, 0.10 mL aliquots were prepared via ^1^H NMR analysis in solutions (final volume 0.70 mL) containing 20 mmol/L HCl, 250 μmol/L TSP, and 10% (*v*/*v*) D_2_O. Immediately thereafter, these analyte solutions were transferred to NMR tubes. The remaining WMSS samples were then equilibrated at 37 °C in water baths, and, subsequently, 0.10 mL aliquots were removed at 60-min intervals prior to preparation via ^1^H NMR analysis, as stated above, ^1^H NMR spectra (600 MHz) were acquired using the parameters described in Section 2.3.

In further experiments designed to address and challenge the use of prior oral rinsing episodes for salivary NH_3_/NH_4_^+^ concentrations, as employed in the studies reported in Refs. [34,35], saliva samples from a single pre-fasted participant were similarly collected each day over a 3-day period, as described in Section 2.1. Following collection of this baseline control sample, additional samples were collected immediately after administration of a bottled spring water-based oral rinse (3 mL for a period of 20 s), and WMS sampling was sequentially continued at 30-min intervals for a total period of 3 h. Samples were rapidly prepared via analysis, as described above, and ^1^H NMR spectra were acquired using the parameters listed in Section 2.3. In this manner, the recovery of salivary NH_4_^+^ ion from these oral rinsing episodes was evaluated.

### 2.8. Sensitivity of ^1^H NMR Analysis of Salivary NH_4_^+^ Ion, and Computation of Signal-to-Noise (STN) Ratios and Lower Limit of Detection and Quantification Values (LLOD and LLOQ Respectively)

Ammonium chloride concentrations of 30, 60, and 125 µmol/L present in the final analyte medium were prepared at a final volume of 0.70 mL containing 20 mmol/L HCl, 250 μmol/L TSP, and 10% (*v*/*v*) D_2_O, as described above. Samples were placed in 5-millmetre diameter NMR tubes and acquired using the ROBUST-5 pulse sequence with either 128 or 1024 scans and the pre-set parameters listed in Section 2.3. STN ratios were estimated via the root mean square of the average noise level, and the peak height intensity of the central line of the NH_4_^+^ ion triplet (δ = 7.08–7.14 ppm) was measured for each spectrum acquired using Jeol Delta version 5.3.1 software (Jeol UK, Welwyn Garden City, UK).

### 2.9. Statistical Analysis of Experimental Data

Computation of descriptive statistics was achieved using GraphPad prism version 9.0 software, as was the performance of linear regression calibration analysis and one-way analysis-of-variance (ANOVA) applied to experimental datasets. Comparisons of differences between the standard calibration curve and SAM methods for the ^1^H NMR analysis of WMSS NH_4_^+^ ion was performed via a paired sample t-test (XLSTAT2020 software option, Addinsoft, Paris, France). This software was also used to perform a two-way randomised blocks ANOVA model to determine the significance of participant- and gender-based contributions to these WMSS NH_4_^+^ levels.

## 3. Results

### 3.1. Detection of Ammonia as Ammonium Ion (NH_4_^+^) in WMSS Samples

The NH_3_/NH_4_^+^ base/acid system has a pKa value of 9.2 [36], and, therefore, for biofluid samples at physiological pH values (e.g., ‘resting’ human saliva at pH 7 and blood plasma at pH 7.4 [8,32]), the protonated NH_4_^+^ form is the dominant species. Azagra et al. [5] successfully detected and monitored NH_4_^+^ ions in human plasma using ^1^H NMR analysis at pH values of ≤3.5, with an optimal pH value of 2, and their results identified a NH_4_^+^ ion triplet signal (of 1:1:1 line intensity) with the highest signal-to-noise (STN) ratio observed. We, therefore, elected to investigate the identification and quantification of NH_4_^+^ in WMSS samples using ^1^H NMR analysis before and after adjusting the sample medium to similar levels of acidification.

Typical ^1^H NMR spectra obtained using a WMSS sample following 1/7 dilution at pH values of 6.95 or 2 are displayed in Figure 1, with the latter value obtained following the addition of HCl. Analysis of the 6.90–7.30 ppm region shows that at a pH value of 2, ^1^H NMR-detectable levels of NH_4_^+^ were observed, which was confirmed based on the appearance of the NH_4_^+^ ion 1:1:1 intensity triplet centred at δ = 7.08–7.14 ppm (brown trace); this resonance was completely absent from the spectra acquired for pH values at or very close to neutrality. Low levels of deuterated isotopomers were also observable in these spectra upon the addition of 10% (*v*/*v*) D_2_O. However, the restriction of D_2_O contents added to this content level did not present any major problems regarding the electronic integration of resonance intensities.

The removal of this splitting in NH_3_ itself is ascribable to the more rapid relaxation of the ^14^N nucleus arising from the quadrupole mechanism, which takes place due to the greater electric field gradient in the case of surroundings with low symmetry; however, an enhanced exchange rate of the ^1^H nuclei remains a possibility [37]. Moreover, Sanders et al. [38] describe the ^1^H NMR profiles of ammonium ion isotopomers, which display deuterium coupling splittings at high digital resolutions.

Since NH_4_^+^ is tetrahedral and much more symmetric than NH_3_, the observation of its ^1^H NMR signal via the ^1^H-^14^N scalar coupling pattern is only possible in view of its highly symmetric environment. The ^1^J-^1^H-^14^N scalar coupling constant for NH_4_^+^ ion was 52.36 Hz in our acidulated aqueous system, which is a value fully consistent with a previously determined value of 52.3 [29]. Although the ^1^H-^15^N and associated partially deuterated isotopomers are, indeed, present, the natural abundance of ^15^N is much lower than 1% of that of ^14^N; therefore, these ammoniacal resonances and their coupling patterns remain beyond the limit of detection for this technique.

Spectra acquired via the application of the ROBUST-5 pulse sequence [31] had much higher intensity values for the NH_4_^+^ ion triplet signal than those obtained using the standard 1D *noesy-presat* option, which is commonly employed in NMR-based metabolomics protocols [8,29], as previously observed for the analysis of aqueous solutions of urea [10]. This observation was highly reproducible in the experiments that we conducted.

Others factor clearly visible in these pH-dependent spectral profiles, which were acquired via the same diluted WMSS samples, were resonances assignable to a range of further endogenous metabolites, including largely microbiome-sourced organic acid anions, such as propionate, acetate, pyruvate, succinate and formate, and amino acids (e.g., glycine and alanine), as well as further biomolecules, including choline and guanidoacetate. As expected, the chemical shift values of a number of these resonances significantly shift upon reducing the pH value from neutrality to 2: these values include downfield and upfield shifts observed for resonances of acetate and formate, respectively, with this shift being attributable to protonation in their carboxylate functions. A full listing of the chemical shift values and coupling patterns of signals visible in the ^1^H NMR of WMSS samples is provided in Ref. [8].

### 3.2. Sensitivity of the ^1^H NMR Analysis of NH_4_^+^ in WMSS Samples at pH 2

**Figure 2:**
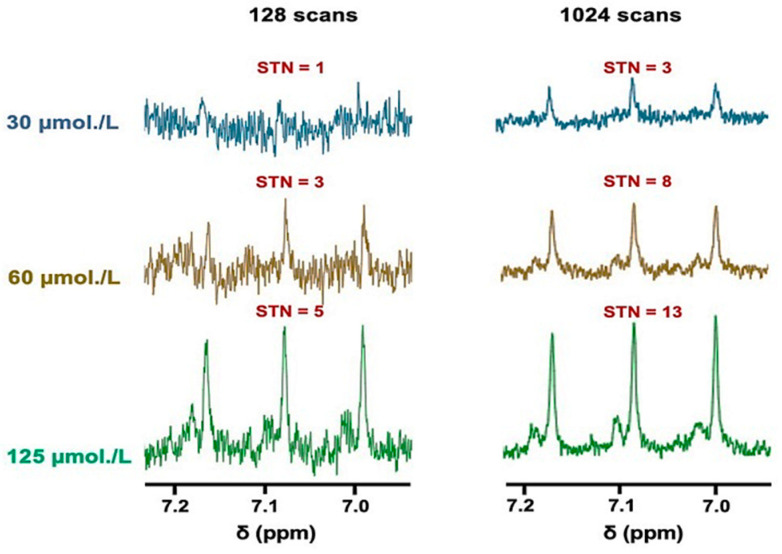
The sensitivity of NH_4_^+^ ion determinations via ^1^H NMR analysis. ^1^H NMR spectra of NH_4_^+^ ions present at standard concentrations of 30, 60, and 125 µmol/L in aqueous solutions containing 20 mmol/L HCl (pH 2) and 10% (*v*/*v*) D_2_O, with either 128 (**left panel**) or 1024 (**right panel**) scans conducted; these concentrations correspond to the final levels of all analyte solutions. The figure also shows the STN ratio of the central line of the NH_4_^+^ ion signal for each spectrum acquired.

The sensitivity of the technique employed for this analyte was determined using either 128 or 1024 scans, with the former type representing a standard for many ^1^H NMR metabolomics investigations (Figure 2 and Figure S1, the latter in the Supplementary Materials section). Clearly, the plots of STN ratio versus standard calibration NH_4_^+^ ion concentration (added as NH_4_Cl) in spectra acquired using either 128 or 1024 scans showed strong linear relationships between these variables throughout the concentration range evaluated.

An acquisition of 128 scans reliably gave a LLOD value of 60 µmol/L NH_4_Cl, which had a corresponding STN parameter of 3. However, reliable quantification of salivary NH_4_^+^ ions requires a STN value of 10, which indicates that minimal concentrations of 200 µmol/L would be necessary to reliably detect this biomarker using this method. However, as expected, increasing the number of scans to 1024 showed an approximately 3-fold improvement in the STN ratio, achieving a resulting LLOD value of only 30 µmol/L and a reliable LLOQ threshold of approximately 80 µmol/L (Figure 2).

### 3.3. Investigations of Hydrogen-Deuterium Exchange and Quadrupolar Splitting of the NH_4_^+^ Ion ^1^H NMR Resonance

**Figure 3:**
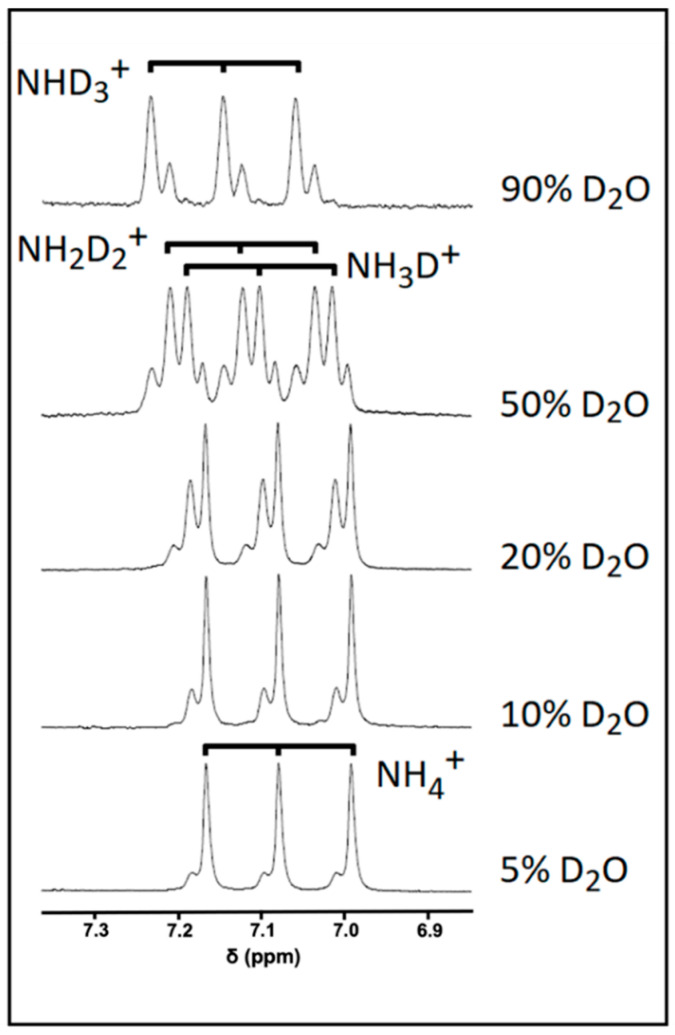
The effect of added D_2_O concentration on the NH_4_^+^ ion resonance pattern. ^1^H NMR spectra of a 5 mmol/L NH_4_^+^ ion calibrant solution with increasing added D_2_O concentrations (from 5 to 90% (*v*/*v*)) in the presence of 20 mmol/L HCl (final pH 2).

A significant concern when attempting to directly quantify metabolites containing exchangeable protons arises from their ability to exchange with deuterium from D_2_O. Indeed, this exchange process can give rise to signals that are significantly under-represented compared to their non-exchangeable counterparts. This issue is shown to be particularly evident by analysing the NH_4_^+^ exchange profile with deuterium from D_2_O added in aqueous acidic solutions that contain increasing volume contents of this deuterated NMR solvent (Figure 3). In a 5% (*v*/*v*) D_2_O solution, the ammonium ion 1:1:1 ^1^H NMR triplet signal was further split into both major and minor composite lines, albeit only to a minor extent. Increasing the concentration of D_2_O to 10% (*v*/*v*) gave rise to a relative increase in the intensity of the more minor lines expressed relative to that of the major signal, and at contents of >10% (*v*/*v*), further new resonance splitting lines appeared. Indeed, at a level of 50% (*v*/*v*) D_2_O, each NH_4_^+^ ion signal is split into four clear lines with an approximate intensity of 1:3:3:1, which is an observation consistent with the formation of each of the four visible isoforms of this analyte following deuterium exchange: NH_4_^+^, NH_3_D^+^, NH_2_D_2_^+^, and NHD_3_^+^. Further increases in added D_2_O content favours increases in the formation of highly deuterium-substituted isoforms, such as NHD_3_^+^ and ND_4_^+^, with the latter isoform being completely ^1^H NMR-invisible. However, the presence of ND_4_^+^ cannot be ruled out, even at levels of 5 or 10% (*v*/*v*) of added D_2_O, with the latter figure representing a level at which most water-based ^1^H NMR-based metabolomics studies are performed. Therefore, at least part of the full NH_4_^+^ ion signal may not be represented in a ^1^H NMR profile in view of the presence of this fully deuterated isoform; hence, without the implementation of a correct series of calibration standards, a direct quantification protocol would yield erroneous underestimates of NH_4_^+^ ion concentrations in biofluids or alternative samples used for analysis.

### 3.4. Establishment of a Reliable Standard Calibration Curve Method to Determine NH_4_^+^ Ion Concentrations in WMSS Samples

**Figure 4:**
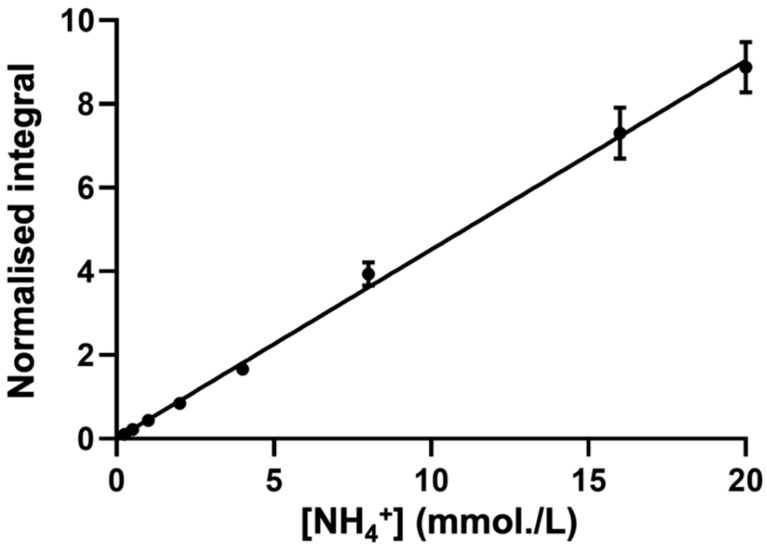
The typical calibration curve for NH_4_^+^ ion determinations. The calibration curve of NH_4_Cl standard solutions (r = 0.9891) generated through successive additions of NH_4_Cl in aqueous solutions that contain a final concentration of 20 mM HCl (final pH 2) and 10% (*v*/*v*) D_2_O. TSP-normalised integrals of the central line of the NH_4_^+^ ion signal were plotted as functions of the standard ammonium chloride concentration. Error bars indicate ± SEM values.

A typical calibration plot that shows the TSP-normalised integral of the central NH_4_^+^ ion signal (δ = 7.08–7.14 ppm) as a function of known standard NH_4_^+^ ion concentration is shown in Figure 4.

To ensure that the standard curve approach generated a reliable means of the determination of WMSS NH_4_^+^ ion concentrations, the standard curve-calculated concentration of a series of validation samples of previously unknown concentrations of NH_4_^+^ added as ammonium chloride (10, 20, and 40 mmol/L) were determined in order to check for bioanalytical accuracy and consistency. From these analyses, deviations from the known, pre-calibrated concentration values were +6.40, −0.75, and −3.82% only, respectively, and, therefore, the observed maximal deviation was only ±6% of the actual known values of these calibration standards. These observations confirm the reliability of this standard curve methodology in determining unknown salivary concentrations of NH_4_^+^ ion.

For all determinations, in order to minimise the overall buffering capacity contributions of WMSSs [39], a 1/7 dilution of these samples was employed to ensure that the pH values of both of the standards, as well as those of the analyte samples, remained constant at 2. In view of the close agreement between WMSS sample NH_4_^+^ ion levels estimated via the ^1^H NMR-based standard linear calibration and SAM methods applied (as described in Section 3.5 below), together with its reliability and high throughput ability, we elected to employ the former method to determine the concentrations of salivary NH_4_^+^ ions in *n* = 27 individual samples collected from a total of 7 healthy (control) participants following a minimum of 8 h of fasting/oral environment activity abstention (Table S1), giving a mean ± SD concentration of 11.39 ± 4.47 mmol/L (range 2.99–18.52 mmol/L) in this cohort. Statistical analysis performed via a randomised blocks ANOVA model (albeit without a first-order interaction effect being considered) found that there were significant differences ‘between-participants’ (*p* = 0.046), with one participant giving levels higher than those of other participants, but not ‘between-genders’. However, there was also no clear or significant relationship between these salivary analyte levels and the age of the participants.

### 3.5. Application of the Standard Addition Method (SAM) to Confirm Estimated Salivary NH_4_^+^ Ion Concentrations

**Figure 5:**
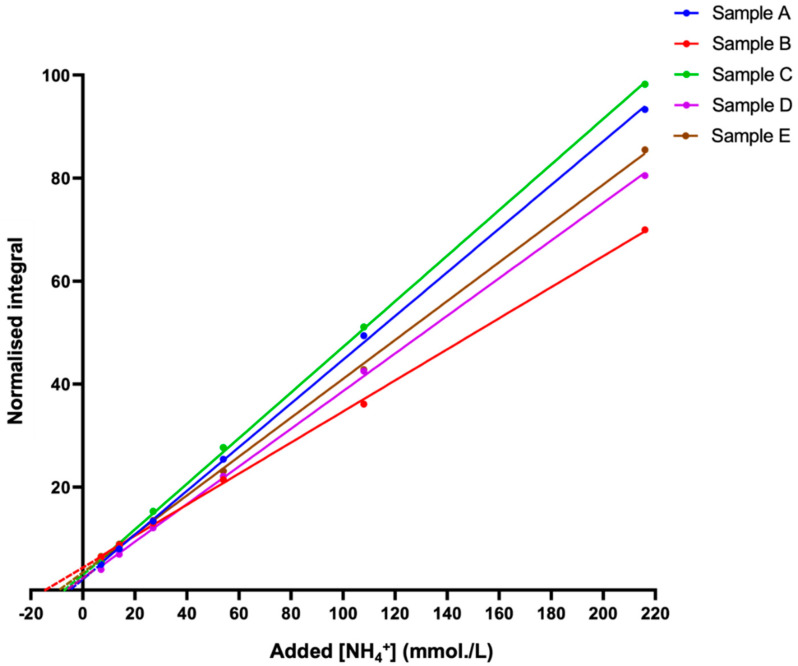
The standard addition method (SAM) plots for NH_4_^+^ determinations in human WMSS samples. The standard addition method (SAM) curves were generated via successive additions of an NH_4_Cl standard stock solution to five different batches of saliva samples collected from healthy participants who were pre-fasted for a period of ≥8 h prior to sample collection. TSP-normalised integrals of the central line of the NH_4_^+^ ion triplet signal (in 10% (*v*/*v*) D_2_O) were plotted as a function of the added ammonium chloride concentration. The samples contained a final concentration of 20 mmol/L HCl (pH 2). R^2^ values for these linear relationships ranged from 0.9994 to 0.9997.

The standard addition method (SAM) is a valuable indirect approach that was used to determine or confirm the concentration of analytes, such as metabolites, in a given sample [40]. A major advantage of this technique is that it bypasses all of the problems described above regarding direct quantification, since deuterium exchange, as well as the pulse sequence employed, will remain exactly equivalent for both the unknown sample and the authentic calibration standards, an advantage that negates their potential influence on the final determined concentration values. Furthermore, with the addition of pre-set volumes of analyte standard calibration solutions to an unknown sample, the sample matrix environment in which the standards are added remains constant, which is a process favouring concentration determinations in unknown samples that are of a high level of complexity, e.g., the WMSS samples analysed in this study. We, therefore, employed this approach to determine the levels of NH_4_^+^ ions present in five typical batches of fasted WMSS samples collected from healthy individuals, and this goal was achieved by integrating the central NH_4_^+^ ion signal line (δ = 7.08–7.14 ppm) as a function of the added NH_4_Cl concentration (Figure 5).

However, differences between the slopes of the plots shown in Figure 5 are potentially explicable based on commonly observed matrix effects of the SAM technique [40], with these effects potentially arising from marked differences between the multicomponent metabolite compositions, particularly NH_4_^+^-binding macromolecules (e.g., proteins) and NH_3_-complexing metal ions (e.g., Ca^2+^ and Mg^2+^), present in WMSS samples [26], along with differences between the NMR-sensitive physicochemical parameters, such as analyte medium ionic strength (I) values and viscosities [8].

Taking into consideration the dilution within the ^1^H NMR analysis samples, and following extrapolation of the linear regression SAM models to the abscissa axis, the concentrations of NH_4_^+^ ions in each of the five samples investigated were computed and directly compared to those estimated via the standard calibration plot method (Figure S2). With the exception of one sample, which deviated by as much as 19%, all analysed samples were located within an acceptable level of agreement. Excluding this sample, the mean ± SD percentage deviation between the two methods was 5.84 ± 5.90%, the magnitude of this small difference being not at all significant when analysed using a paired sample t-test.

In view of the good agreement between the direct calibration curve and time-consuming SAM approaches used, it appears that the former approach is not affected by the potential above-considered limitations.

### 3.6. Attenuation of Salivary NH_4_^+^ Concentration by the Pre-Sampling Administration of an Aqueous oral Rinse to Participants, and Its Recovery Therefrom

Since previous reports of salivary NH_3_/NH_4_^+^ ion levels involved investigations featuring the administration of water-based oral rinses prior to saliva sample collection [34,35], this pre-sampling approach was also explored here in order to evaluate its effect on salivary NH_4_^+^ concentrations. For this purpose, these experiments were focused on a sequential time-series of samples collected from a single WMS donor, with time-dependent sample collections replicated over three separate days. These studies were limited to a single healthy control sample donor in order to avoid potential complications arising from the ‘between-participant’ source of variation.

**Figure 6:**
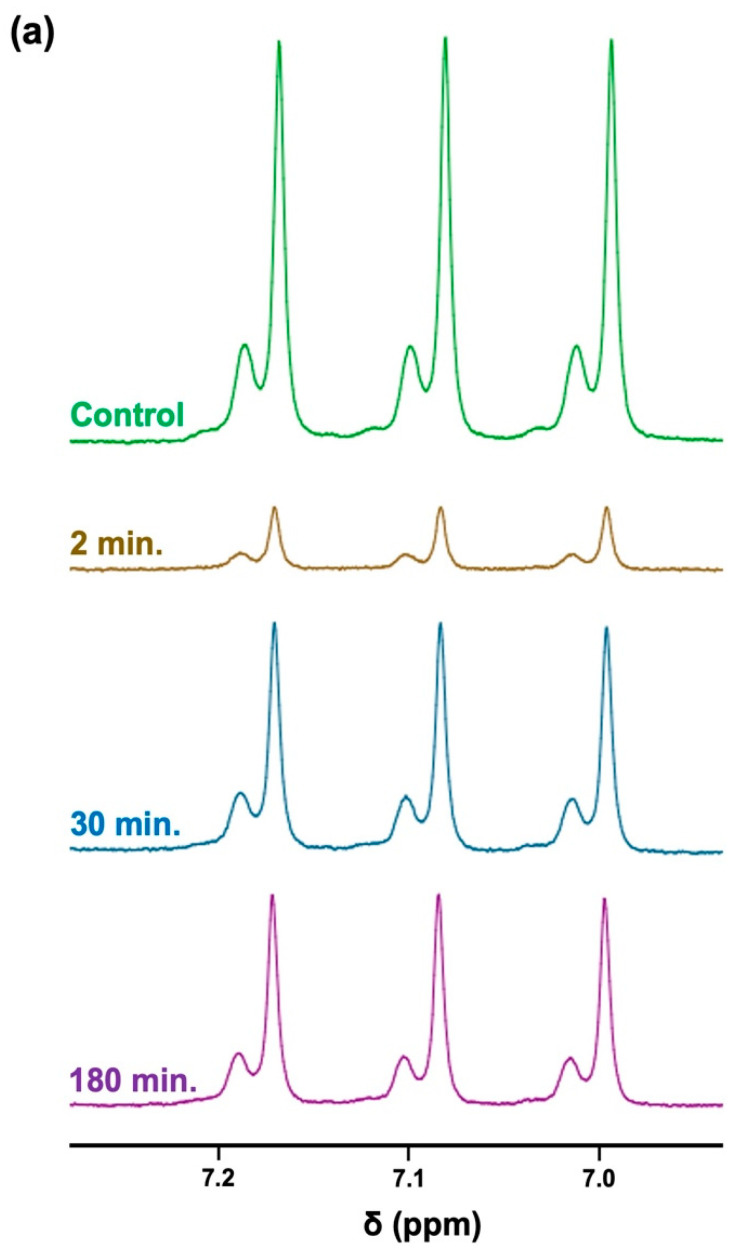

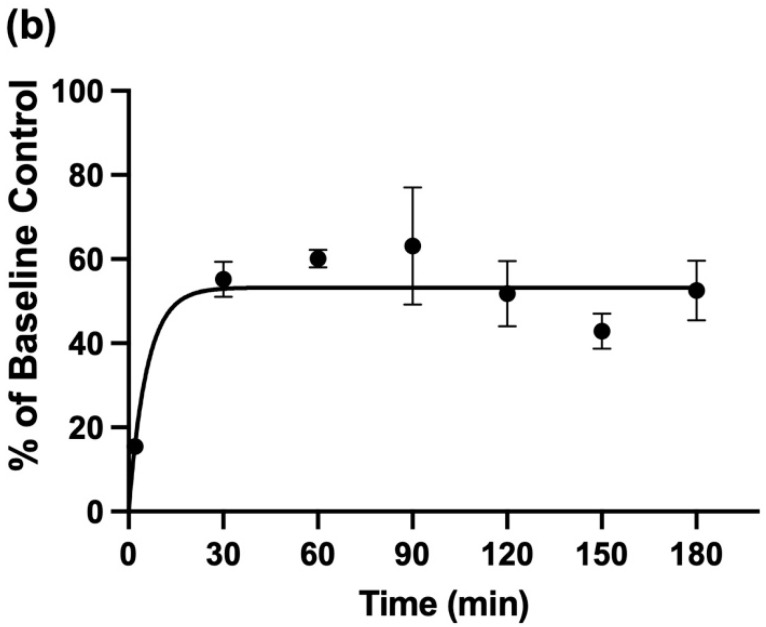
The time-dependence of the recovery of salivary NH_4_^+^ ion concentrations following the administration of an aqueous oral rinse. (**a**) ^1^H NMR spectra of NH_4_^+^ ions following the 8-h fasting/oral activity abstention period protocol (mean NH_4_^+^ level 18.6 mmol/L) and sequentially at 2, 30, and 180 min following the use of an oral-rinsing episode with bottled water. The resonance intensities were normalised to those of the TSP internal standard. The typical spectra are shown. (**b**) The plot of the mean(±SEM) percentage of the original mean pre-rinsing basal control NH_4_^+^ concentration versus time subsequent to the oral rinsing episodes conducted.

The ^1^H NMR profiles shown in Figure 6a display results acquired from these experiments, which clearly demonstrate highly significant decreases in TSP-normalised NH_4_^+^ ions’ ^1^H NMR signal intensities up to 90 min following this single oral rinsing protocol, with a very substantive reduction being observed after a further 2 min. Additionally, a plot of the mean ± SEM percentage of the salivary baseline concentration of NH_4_^+^ versus post-oral rinsing time point is displayed in Figure 6b. Clearly, the major decrease in the mean salivary NH_4_^+^ concentrations observed at only 2 min post-fasting was 15% of this basal value. Subsequent monitoring of this mean level at the 30, 60, 90 120, 150, and 180-min post-rinsing time points demonstrated that although, at the 30-min time point, it returned to roughly 50–60% of its pre-mouth-rinsing control value, this value was found not to significantly rise again for up to a further 180 min.

### 3.7. Time-Dependence of Salivary NH_4_^+^ Ion Concentration in WMSS Samples following Sample Collection and Laboratory Processing: Influence and Suppression of Urease Activity

Urea is converted to ammonia through the action of the bacterial enzyme urease, which represents one key contributor to the generation of this agent in biofluids. Unfortunately, this process may hinder the accurate determination of NH_4_^+^ ion in this biofluid, since this transformation may be occurring immediately from the point in time at which samples are first collected from host donors, and, therefore, NH_4_^+^ ion concentrations may significantly alter during subsequent laboratory processing and storage episodes. Indeed, one study conducted in the 1970s concluded that the majority of salivary urea are converted into NH_3_ ions within 290 min following collection and incubation at 37 °C, albeit with increases in the level of this analyte being observed within the first 100-min equilibration period [7]. This suggestion is of particular concern when conducting NMR-based studies, since sample preparation following collection may vary from sample to sample, and this issue may adversely influence the final levels of NH_4_^+^ ions that are determined; hence, the time of ^1^H NMR analysis following sample collection may serve as a confounding variable for consideration. We, therefore, investigated the rate of formation of NH_4_^+^ ions in WMSS samples (*n* = 3) using ^1^H NMR analysis involving electronic integration of the central coupling line of the NH_4_^+^ ion triplet (δ = 7.08–7.14 ppm) at increasing equilibration time points and at a temperature of 37 °C following sample collection (Figure 6). The results acquired indicated that there appeared to be no significant increase in salivary NH_4_^+^ ion levels within the first 2–3 h of equilibration, though a rise was, indeed, observed at later time points, with a highly significant increase observed following an 18-h period at this temperature (*p* = 0.002). These results indicated that the post-sample collection rate of NH_3_ (analysed as NH_4_^+^ ion) formation, which presumably stemmed from the actions of urease on urea in these WMSS samples, was slower than that previously reported by Kopstein and Wrong [7]. This result suggests that the 20–30-min time period required for sample preparation conducted at ambient temperature in our studies would not significantly affect the levels of NH_3_/NH_4_^+^ ions determined in solution following WMS sample collection at the baseline zero control time point, an observation that enables researchers to negate the putative post-collection time-dependence of salivary NH_4_^+^ ion concentrations. Indeed, the adjustment of the pH value of analyte solutions to a value of 2, which is completed within a 10–20-min post-collection ‘window’ in our experiments, will serve to completely inactivate the actions of salivary urease, along with those of any further NH_3_-generating enzymes present in this biofluid. Furthermore, with the exception of the centrifugation step performed at 4 °C, all laboratory processing tasks of WMS samples for NH_4_^+^ ion analysis conducted in our laboratory were performed at ambient temperature (roughly 23 °C), rather than at 37 °C.

**Figure 7:**
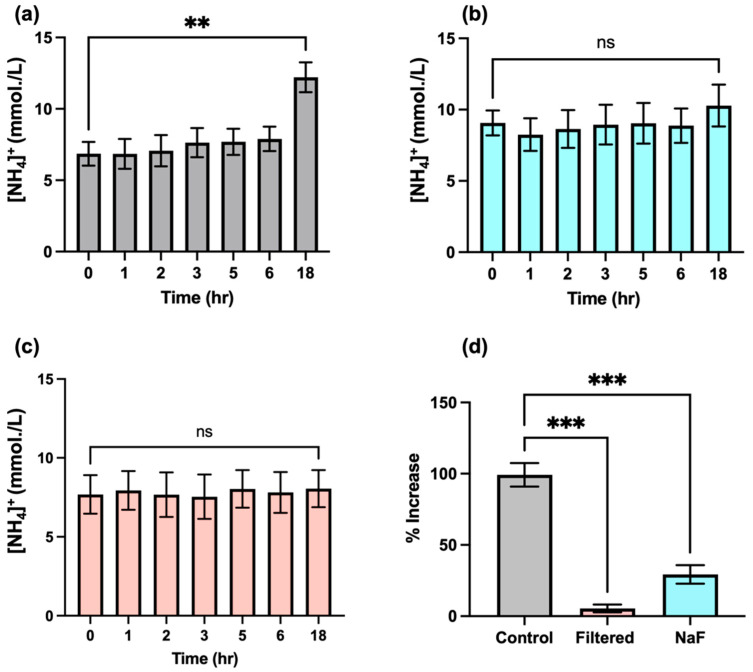
The time-dependent modifications in salivary levels of NH_4_^+^ ions following sample collection. The data shown are mean ± SEM values of ^1^H NMR-determined NH_4_^+^ ion concentrations as functions of time after WMS sample collection for a total of *n* = 3 separate WMSS samples equilibrated at 37 °C prior to analysis, according to Section 2.7. (**a**) Control (untreated) samples (*n* = 3); (**b**) as (**a**), but for separate samples (*n* = 3) following treatment with the urease inhibitor sodium fluoride (final added level 5 mmol/L) immediately after collection; and (**c**) following ultrafiltration immediately post-collection of an additional set of *n* = 3 WMSS specimens using a 3K molecular mass cut-off ultrafiltration system for protein removal. (**d**) Percentage increases observed between the mean 18- and 0-h sampling time points for the zero pre-equilibration control in the fluoride-treated and ultrafiltered sample groups. Abbreviations: ns, not statistically significant. ** indicates *p* = 0.002; *** indicates *p* = 0.0002 (one-way ANOVA).

Additional experiments were also conducted in order to further substantiate the lack of interference of salivary urease and, perhaps, other NH_3_-generating enzymes during the sample preparation stages of our bioanalytical investigations. Firstly, *n* = 3 separate pre-fasted (8-h duration) WMSS samples collected from the same participant (total volume *ca.*5 mL) were treated with the urease inhibitor sodium fluoride (final level 5 mmol/L), and salivary NH_4_^+^ concentrations were determined as functions of time during their equilibration at a temperature of 37 °C to evaluate any modifications to them (Figure 7b). These data clearly show that while a highly significant increase in NH_4_+ ion concentration was observed in WMSS samples at the 18-h time point in untreated samples (Figure 7a), those samples pre-treated with fluoride prior to equilibration resulted in no such augmentation, an observation indicating that urease, perhaps together with further NH_3_-generating enzymes, was satisfactorily inhibited by added fluoride ion. The ‘between-equilibration time’ effect was not found to be statistically significant for these experiments (ANOVA).

An additional protocol involved ultrafiltration of *n* = 3 separate WMSS specimens (once again, all specimens were collected from a single participant) using 3kDa cut-off ultrafiltration devices was performed prior to equilibration at 37 °C and, once again, the time-dependence values of their NH_4_^+^ levels were monitored following this treatment. Results acquired are shown in Figure 7c, and these results, once again, demonstrated that this urease-removing strategy effectively curtailed the rise in salivary NH_4_^+^ concentrations observed without this treatment (as in Figure 7a). Once again, no significant differences were found between the 0-h control and 18-h samples’ equilibration times (ANOVA).

As might be expected, there were highly significant differences found for percentage increases in mean NH_4_^+^ ion levels between the 0- and 18-h equilibration time points of the control group of samples, which were greater than those observed for groups that were either fluoride treated or ultrafiltered prior to equilibration at 37 °C (*p* = 0.0002 in both cases). Therefore, these data suggest that such precautionary routes for the suppression or removal of the activity of urease, as well as, perhaps, activities of related NH_3_-forming enzymes, were both sufficient for removing any potential interferences stemming from these sources during the laboratory processing of WMSS samples for NH_4_^+^ ion analysis, even if the process was prolonged for up to 18 h. However, these protective approaches are not necessary if our protocol for the preparation of these samples, which involves only a 20–30-min duration, and urease-inactivating acidulation of the analyte medium to pH 2 is scrupulously followed.

## 4. Discussion

### 4.1. Evaluation of the ^1^H NMR Method Developed for the Determination of Salivary NH_4_^+^ Ions and Its Potential Limitations

To date, full studies involving the ^1^H NMR-based quantification of NH_3_/NH_4_^+^ ions in human saliva, specifically WMSS samples, have not been reported, although its determination via this technique poses some challenges in view of the exchangeable ^1^H nuclei of this analyte in aqueous solution. A similar approach was applied in an eloquent study performed by Preece and Cerdan in 1993 [29], who applied ^1^H NMR spectroscopy to the analysis of acidified biofluids (blood plasma and urine) collected from experimental rats for the purpose of monitoring the ^15^N to ^14^N ratios of NH_4_^+^ ions present in these samples, and, hence, determining the extent of ^15^N-labelling in these animals (^15^N was infused into them as a ^15^N-enriched ammonium acetate). These researchers concluded that the method developed compared favourably to other strategies employed for the monitoring of ^15^N- labelling, especially because ^1^H NMR analysis has a much greater sensitivity than both ^15^N and ^14^N NMR analyses.

There are two main strategies via which metabolites can be successfully determined through ^1^H NMR analysis using quantitative NMR (QNMR) techniques: the direct method based on the use of an internal standard (usually TSP for metabolomics studies) and the indirect method that uses calibration standards; the latter method is applied in the current study. In the direct method of quantification, a ratio is calculated, which considers the signal integral and the number of protons of both the signal of interest and the internal standard of known concentration (or, alternatively, one that is isolated as a standard calibration solution in an NMR tube-inserted capillary system, meaning that it represents an ‘external-but-internal’ standard). This approach is a high-throughput method that has been used to simultaneously determine the concentrations of many known metabolites. However, a major disadvantage of this approach arises when the metabolite of interest contains protons that are exchangeable with deuterium from pre-added D_2_O, such as those in NH_4_^+^ ions, and this process may give rise to concentrations that are erroneous.

Therefore, for direct the determination of NH_4_^+^ ion concentrations, the pulse sequence involved must be carefully selected, since this sequence can exert a direct impact on signal intensities. In these circumstances, standard pre-saturation techniques are not usually recommended, since the long duration of the pulse designed to saturate protons arising from excessive contents of water would also saturate exchangeable >NH protons, a process that is ascribable to exchange occurring during pulse duration. This process was previously noted for the exchangeable -CO-NH_2_ protons of urea, in which pre-saturation using the 1D noesy-presat sequence attenuated its broad signal located at δ = 5.79 ppm, whereas pulse sequences, such as WATERGATE and WET, exert less of an impact [30]. A similar observation was made for the exchangeable NH_4_^+^ protons when comparing the standard metabolomics noesy-presat sequence with the ROBUST-5 option, with the latter option reportedly not attenuating exchangeable proton signals, albeit with a much more effective suppression of the water signal [31]. In contrast to the noesy-presat sequence, spectra acquired using the ROBUST-5 technique were found via reproduction to have a markedly greater intensity for the NH_4_^+^ ions’ triplet signal, which supports its use as a means of directly quantifying this species; it is possible that further metabolites also contain such exchangeable protons [10].

In addition to using an appropriate pulse sequence, meaning that the NH_4_^+^ ion signal is not being artificially attenuated beyond the threshold point of its quantification (LLOQ), choosing the correct NMR solvent in which the biofluid sample is analysed can also exert a significant impact on the calculated concentration of a metabolite containing exchangeable protons. With NH_4_^+^ ions in aqueous solution, increasing the concentration of D_2_O gave rise to the formation of deuterated isoforms of NH_4_^+^ via a rapid exchange in its ^1^H nuclei with those of deuterium, one of which is completely ^1^H NMR-invisible in the spectra acquired (specifically ND_4_^+^), as shown in Figure 3. Therefore, solvent exchange with added D_2_O also has the ability to artificially suppress the NH_4_^+^ ions’ signal intensity, which will most certainly directly impact the calculated analyte concentration. However, at an added D_2_O solution content level of only 10% (*v*/*v*), as used in the current study, this effect was found to be minimal, with little or none of the ^1^H NMR-invisible ND_4_^+^ species being formed.

One approach for circumventing the potential interference of deuterated isoforms of NH_4_^+^ ions would be to use a non-exchangeable deuterated solvent system to provide the frequency lock during ^1^H NMR acquisition, instead of D_2_O, and selected studies have, indeed, combined this measure with the use of hexa-deuterated dimethylsulphoxide (DMSO-d6) to achieve this result [5,27,28]. However, the disadvantages of such an approach are that undeuterated forms of this solvent, which are known to contaminate commercially available fully deuterated products available, give a large residual resonance at roughly 2.6 ppm (which we find often exceeds a concentration of 3 mmol/L, although it is source dependent), which could potentially superimpose on and interfere with other resonances arising from common biofluid metabolites, such as those of the −CH_2_CO_2_^-^ protons of citrate. In some cases, this residual partially undeuterated DMSO contribution may be the largest signal in the spectra acquired, and, therefore, it could also exert an effect on the receiver gain during NMR acquisition, a phenomenon that results in poorer quality spectra. Moreover, for Ref. [5], which reports on a study focused on the ^1^H NMR determination of NH_4_^+^ in human blood plasma, this biofluid was lyophilised prior to reconstitution in an equivalent volume mixture of both DMSO-d6 and undeuterated DMSO; hence, the above potential limitations may be substantially amplified. Additionally, a further disadvantage is the lyophilisation step required for the extraction protocol featured in this work, which may give rise to the loss of, perhaps, significant amounts of volatile NH_3_, despite performing this process at acidic pH (pH values were adjusted with added trifluoroacetate). Therefore, other approaches that can bypass some of the issues described here would, perhaps, be more suitable when attempting to quantify solution levels of NH_4_^+^ ions via ^1^H NMR analysis.

In view of these observations, the use of appropriate standard calibration curves serves as a valuable approach when quantifying levels of NH_4_^+^ ions, since the effect of the pulse sequence employed and solvent exchange processes may be abrogated, provided that spectra of unknown analyte samples are acquired under exactly the same conditions/parameters as those of the standards. This approach was, indeed, the case when generating the calibration plot shown in Figure 4. For example, a difference in pH between the sample in question and that of the standards can potentially alter solvent exchange, therefore affecting the intensity of the NH_4_^+^ ions’ triplet resonance, as previously reported [5]. In these studies, maintenance of a consistent pH value between the standards and the WMSS sample is rendered complicated, since there is no buffering agent available that can reliably ensure a pH value of 2. Since human saliva exhibits some buffering capacity [39], for our studies, the pH values of both the sample and the calibration standards were carefully modulated accordingly, as was the concentration of HCl added, meaning that they remained equivalent.

Differences observed between the gradients of the SAM plots shown in Figure 5 are probably attributable to previously described matrix effects, which have been reported for the analysis of metformin in post-mortem blood samples via liquid chromatography electrospray-tandem mass spectrometry (LC-ETMS) [40]. Nevertheless, in the current study, a comparison of results from both the standard calibration curve and the SAM approaches on identical WMSS samples yielded very similar estimated levels of NH_4_^+^ ion concentrations that were very positively correlated, as was expected (Figure S2).

Using microdiffusion, Kopstein and Wrong [7] determined that oral saliva contains NH_3_ at levels varying from 0.60 to 26.00 mmol/kg in both healthy and uraemic subjects, with mean concentrations of 3.8 and 13.6 mmol/kg for these two classes of participants, respectively, although the former value was only estimated via an analysis of *n* = 4 subjects (albeit with *n* = 8 uraemic patients). The parotid saliva samples collected, however, contained little or none of this analyte. Moreover, our estimated WMSS NH_4_^+^ concentrations for healthy participants ranged from 2.99 to 18.52 mmol/L for *n* = 27 WMSS samples (mean level 11.39 mmol/L). The lower concentrations of this analyte found in the healthy control cohort of the Ref. [7] study were, however, similar to those reported in the scientific literature: for example, a study using the micro-PAD detection technique only estimated a mean level of 2.48 mmol/L. in healthy control subjects [34]. Furthermore, other studies using an established spectrophotometric method involving the derivatisation of NH_3_ to a blue-coloured indophenol product (this stage involving its reaction with phenol and hypochlorite anion) [35] or a flow-based ion-selective electrode approach [41] yielded mean salivary concentrations of 4.4 and 7.7 mmol/L, respectively. Indeed, these apparent discrepancies between the results of our study and those previously reported [34,35,41] are very likely explicable based on differences in saliva sample collection procedures. For example, in the micro-PAD study reported in Ref. [34], healthy human participants were required to rinse their mouths with water at least 1 h prior to sample collection. Similarly, in the method described in Ref. [35], an initial mouth-rinsing substance was provided to healthy participants for use before sample collection, although an exact time period prior to saliva sampling was not specified in that report. Hence, for these investigations, the initial mouth rinse purging episodes very likely acted as significant diluents for salivary metabolites, including NH_4_^+^. Furthermore, the minimal oral environment abstention duration period adopted prior to sample collection may be insufficient for levels of NH_3_/NH_4_^+^ to re-equilibrate back to their basal control levels. Consistent with this fact, our results, as shown in Figure 6, clearly demonstrate that a protocol involving a pre-sampling oral-rinsing episode is sufficient to cause a very major attenuation of WMSS NH_4_^+^ concentrations; therefore, we strongly suggest that for future studies, appropriate precautions are taken to avoid using this mouth-rinsing approach in order to attain acceptable and realistic levels.

Furthermore, in Ref. [41], salivary NH_4_^+^ ion levels were compared between healthy and CKD subjects, yet samples were collected only 1 h following a meal, a time delay that is considered insufficient to avoid complications arising from the introduction of potentially interfering exogenous dietary agents into the oral environment [8]. Conversely, in the current study, samples were collected after a minimum 8-h fasting/oral abstention period, which did not involve any form of oral activity. Although an insufficient oral abstention duration may, at least in principle, also contribute to a somewhat higher mean salivary NH_4_^+^ concentration than recorded in studies conducted without this precaution (in view of the entry of NH_3_/NH_4_^+^ ions into the oral environment from food sources), the lower salivary concentrations found in these reports are probably derived from dilutions resulting from the oral-rinsing episodes incorporated into their protocols. This issue, once again, highlights the importance of adopting a suitable fasting/oral abstention period prior to WMS sample collection. These abstention periods are also recommended for the collection of virtually all other biofluids, such as those focused on the lipidomics monitoring of lipoprotein-associated triacylglycerol lipids in blood plasma. Of course, the performance of realistic ‘between-disease’, between-participant’, and ‘between-assay’ comparisons of salivary NH_4_^+^ concentrations would require assurances that all samples are collected and processed in exactly the same manner prior to analysis in order to avoid analytical inconsistencies.

Interestingly, the study reported in Ref. [7] also revealed that saliva isolated from the parotid duct of both healthy control and uraemic participants contained a urea concentration that was 86% of that of the blood plasma level, although in mixed saliva, this value was only 31%. Oral salivary NH_3_ concentrations were found to be positively correlated with those of plasma urea. Moreover, these researchers found that equilibration of mixed oral saliva samples at a temperature of 37 °C for a 290-min period gave rise to the disappearance of urea, with this observation accompanied by a steady increase in salivary NH_3_ levels. These enhancements in NH_3_ concentration were putatively largely ascribable to its liberation from urea via the actions of bacterial urease during the first 100 min at this temperature. However, additional non-urea sources for this analyte were considered important at later equilibration time points [7]. In the current study, however, we found that following our WMS sample collection protocol, especially during the 20–30-min stage involving preparation of these samples for ^1^H NMR analysis (conducted at ambient temperature and 4 °C) immediately thereafter (Section 2.1 and Section 2.2), there were no significant changes in salivary NH_4_^+^ concentrations, and these concentrations remained stable for up to several hours thereafter, even at an equilibration temperature of 37 °C. Following this time point, a rise in WMSS NH_4_^+^ concentrations was observed, with this increase becoming highly statistically significant at an 18-h post-collection time point when exposed to this temperature (Figure 7).

These differences observed are readily explicable based on the following reasons: (1) the much lower temperature of the current study’s laboratory sample preparation stages (roughly 23 °C versus 37 °C in Ref. [7]), along with the quite rapid duration of these episodes (20–30 min.); and (2) adjustment of the final analytical pH value of these samples to 2 (a process that occurs within post-sample collection time points of 10–20 min or less), which will be more than sufficient to inactivate any urease enzyme activity present in the processed WMSS samples [42]. However, our results also show that WMSS-sample NH_3_/NH_4_^+^ ions are retained and do not significantly increase in concentration for up to 2–3 h when incubated at a temperature of 37 °C (Figure 7).

Additional experiments conducted in the current study explored methods for the inhibition or removal of the NH_3_-producing enzyme urease during the laboratory processing of WMSS samples via ^1^H NMR analysis (Figure 7). Indeed, it was found that the employment of the urease enzyme inhibitor fluoride (final concentration 5 mmol/L) prevented the highly significant elevation of this analyte’s concentration within the 6–18-h time zone when samples were equilibrated at 37 °C (the mechanism of this process involves the direct complexation of fluoride anion by nickel(II) ions available at urease’s active centre [33]). Similarly, the prior ultrafiltration of these samples using a device with a 3 kDa cut-off membrane was also found to completely block this 6–18-h increase in salivary NH_4_^+^ level, and taken together, these precautionary measures strongly indicate that this analyte’s upregulation is derived from the urease-mediated catalytic breakdown of WMSS urea, albeit on a time-lagged basis. Other sources of NH_3_ in vivo include the complete catabolism of amino acids to their corresponding energy intermediates, which is a process that generates this toxin [43]. Indeed, amino acids with high ammoniagenic potentials are arginine, aspartate, glutamine, lysine, and methionine.

In addition to the very limited sample size featured in Ref. [7], a further consideration lies in the fact that in this study, patients were, firstly, required to chew an NH_3_- and urea-free gum during sample collection and, secondly, use an oral rinse prior to collection, with only a very limited and insufficient 10-min equilibration delay involved between these sequential episodes. These sample collection criteria will both serve to provide major barriers to the reliable estimation of salivary NH_3_ concentrations, with the second one, once again, potentially providing an oral rinsing-based ‘diluent’ system for this analyte.

An overall literature summary of previously reported salivary NH_3_/NH_4_^+^ concentrations in human healthy control, as well as age-matched study participants with a series of diseases, is provided in Table 1. With the exception of the current study, all reported investigations involved saliva stimulation via the chewing of a paraffin pellet or film and/or the use of a water-based mouth-rinsing regimen at various times prior to sample collection; this method provides a likely explanation for the lower NH_4_^+^ concentrations found in these studies. However, despite this complication, one study [41] found a mean level as high as 7.7 mmol/L for healthy control subjects.

The mean concentration of 4.4 mmol/L (range 1.1–12.1 mmol/L) found in healthy control mixed saliva samples [35] was reported in Ref. [13] to be ca. 150-fold greater than that found in blood. According to the authors, undiluted saliva for NH_3_/NH_4_^+^ analysis could be stored for a period of 14 days at −20 °C, whereas diluted saliva was reported to be stable at 4 °C for only 1 h; after these periods, the level of this analyte increased significantly.

### 4.2. Application of ^1^H NMR Techniques to the Analysis of Alternative, Non-Ammoniacal Biomolecules with Exchangeable Protons

Although ^1^NMR analysis may also be applied to the determination of other biomarkers with exchangeable protons, such as urea present in human saliva and other biofluids, due precautions should be taken in order to avoid complications with the analysis protocols applied. In the case of urea, these issues arise via the exchange in its -NH_2_ protons with deuterium from any D_2_O added to analyte solution media as a field-frequency lock, as well as using protons supplied by the water solvent itself. Also, the time-dependent enzymatic degradation of urea in these analytical matrices following sample collection, which is a process yielding NH_3_ and, therefore, NH_4_^+^ as a product, presents another complication.

Of notable interest, for the pre-treatment of samples with HCl in periods of 10–20 min following their collection in the current study (in order to adjust their pH values to a value of 2), a preparation stage, which was applied for analytically optimised ^1^H NMR detection and monitoring purposes, was a process that also fortuitously limited the loss of NH_3_ in saliva in view of its relatively high level of volatility, even at ambient temperature (boiling-point −33.35 °C). Hence, related sample pre-treatments may also be appropriate for the analysis of alternative biomarker analytes.

Additionally, replacement of the aqueous solution medium with those of non-protic deuterated organic solvents, such as DMSO-d6, may also serve as an analytical advantage in such cases, although careful checks will have to be performed for any loss of volatile agents, such as NH_3_, from analyte media during any lyophilisation stages involved, as well as the presence of any interfering ^1^H NMR signals arising from non-fully deuterated solvent impurities being present, notably DMSO-d5 in the case of hexadeuterated DMSO.

## 5. Conclusions

In conclusion, results obtained from this study confirmed that NH_4_^+^ can be reliably determined in human WMSS samples using a newly developed ^1^H NMR analysis protocol, which involves the acidification of these samples to a fixed pH value (2), followed by the observation of the characteristic NH_4_^+^ ion triplet resonance and electronic integration of its central spectral line. Indeed, the approach adopted is of much value, since the analyte contains exchangeable >N-H protons and, therefore, its analysis via ^1^H NMR spectroscopy, which is a widely employed technique in untargeted metabolomic studies, is rendered complicated. Our results also revealed an acceptable agreement between standard calibration curve and SAM bioanalytical strategies employed, which highlighted their reliabilities when used in this analysis. Additionally, the analysis of a series of unknown assay calibration samples readily confirmed and validated both of these approaches. Moreover, we also verified that the post-collection time required for the laboratory preparation of samples did not interfere with the results acquired (although post-collection increases in salivary NH_4_^+^ level were observed, these increases were not of any significant magnitude until after an equilibration time period of ≥2–3 h at ambient temperature, and which were maximal at 18 h post-collection).

One limitation, which may affect the reliability of the NMR method developed here, is potentially ascribable to the use of D_2_O. Nonetheless, the solution with D_2_O content of 10% (*v*/*v*) employed in this bioanalytical investigation gave rise to only a very minor extent of deuterium substitution, without the detection of significantly interfering levels of higher deuterium-substituted isotopomers, including ^1^H NMR-invisible ND_4_^+^.

Furthermore, the method developed also did not require the use of any costly non-protic deuterated NMR solvents, such as DMSO-d6, which was a critical requirement of the NMR-based assay system used for NH_4_^+^ ion optimised for blood plasma in Ref. [5]. In regard to the sensitivity of our ^1^H NMR analysis method used for determining the concentration of NH_4_^+^ at pH 2, the LLOD and LLOQ values were found to be 30 and 80 μmol/L, respectively, although a total of 1024 scans were required to achieve each figure. Clearly, these values would decrease significantly with the incorporation of lower levels of dilution into the assay system, i.e., higher volumes of analyte solutions, into the final fixed-volume analysis mixture, which represented only a facile adjustment to the analytical protocol.

Use of the methods employed here for use in the ^1^H NMR-based NH_3_ monitoring regimens (as NH_4_^+^ ion) in WMSSs are also applicable to other analytes with exchangeable ^1^H nuclei. These methods include the following approaches: (1) the institution of a reliable pre-validated standard calibration curve and/or the SAM analysis approaches used for this purpose; (2) limiting the D_2_O content of ^1^H NMR analyte samples to minimal levels (e.g., no more than 5 to 10% (*v*/*v*)); (3) the rapid laboratory processing of samples, following their collection, to prevent enzymatic degradation of urea and amino acids, including treatment with acids, such as HCl, or alternative reagents to block their actions; (4) the careful selection of water signal pre-saturation pulse sequences that are to be applied, for example, the use of the ROBUST-5 sequence, since it only exerts minimal effects on exchangeable proton resonances in analyte biomolecules and other agents [31], with this benefit being conferred in the current study (Section 3.1) and confirmed in Ref. [10]; (5) optimisation of the power sources employed for such water signal pre-saturation applications; and, last but not least, (6) the essential incorporation of relevant fasting/oral environment abstention periods prior to sample collection to avoid interferences from any exogenous agents, such as dietary agents with the same molecular identities as those of any biomarkers monitored in biofluids, for example overnight episodes, or a minimum duration of 8 h, as employed in the current study.

Since NH_3_/NH_4_^+^ ions can serve as key biomarkers for a variety of diseases, including CKDs [7] and selected cancers [22], this method could also be readily applied for diagnostics monitoring purposes in a wide range of other disorders. It is also relevant to the oral health research area, hence its determination in oral fluids, such as human saliva, as described here, is of paramount importance. In principle, the bioanalytical protocol developed here may also be applicable to the analysis of other biofluids, including blood plasma and urine, with the former being as described in Refs. [5,29], or appropriate liquid biopsy extracts.

Overall, the standard calibration curve and SAM approaches developed and validated here may also be extended to provide a reliable QNMR system for the monitoring of alternative non-ammoniacal biomarkers with only limited molecular site numbers of exchangeable, but not non-exchangeable, protons available, whether in WMSS or other biofluids (e.g., as described in Ref. [10]).

## Figures and Tables

**Table 1:** Salivary NH_3_/NH_4_^+^ concentrations determined for healthy controls and subjects with a range of diseases, as reported in previously conducted investigations. The table also provides information about the analysis technique/method employed, as well as comments regarding the sample collection protocols involved and results acquired. Abbreviations: n/a, not applicable. * mmol/kg and not mmol/L units; ** mmol/min/μg kinetic units; *** not mmol/L concentration units (none reported in the investigation).

Study Reference	Disorder	Healthy Control Group Mean [NH_3_] or [NH_4_^+^] in mmol/L (±SD, and Range in Brackets, Where Available)	Disease Group Mean [NH_3_] or [NH_4_^+^] in mmol/L (±SD, and Range in Brackets, Where Available)	Analysis Technique/ Method	Sample Collection Procedure
[7]	Uraemia	3.8 *	13.6 *	Enzyme-based microdiffusion method described by Conway [44].	Participants chewed gum and rinsed their mouths 10 min prior to sample collection.
[41]	CKD	7.7	32.9	Flow-based NH_3_-selective electrode.	Saliva samples were collected at least 1 h after eating and tooth-brushing were performed. Mouth rinsing was performed at an unspecified time point prior to sample collection.
[45]	Rheumatoid arthritis (RA)	1.93 ± 0.48 **	3.61 ± 0.76 ** (high disease activity group only)	Enzymatic kinetic/ colourimetric assay.	None specified.
[46]	Dyspepsia	2.57 ± 1.64	2.49 ± 1.26	Indophenol spectrophotometric method.	Saliva was collected 5 min after participants rinsed their mouths with a water-based oral solution. Saliva was stimulated via a paraffin pellet-chewing episode.
[20]	Oral malodour	63 (55–74) ***	Physiological oral malodour: 79.5 (73.8–84.3) *** Periodontal disease oral malodour: 76.5 (68.5–84.8) ***	Flow-based NH_3_-selective electrode.	Each participant was instructed to rinse the oral cavity with 3 mL of distilled water for a period of 10 s before sample collection.
[18]	End-stage renal disease patients undergoing haemodialysis	n/a	3.5 ± 0.3	Indophenol spectrophotometric method.	Saliva samples were stimulated via chewing a paraffin pellet prior to collection.
[47]	Healthy control only	2.2 ± 0.2	n/a	Indophenol spectrophotometric method.	Samples were collected ≥ 2 h after a participant’s last meal. Stimulated samples were collected via chewing a plastic paraffin film for 1 min
[34]	Healthy control only	2.48	n/a	Micro-PAD card technique.	Samples were collected at least 1 h after an oral mouth-rinse was performed with water following a meal.
[35]	Healthy control only	4.40 (1.1–12.1)	n/a	Indophenol spectrophotometric method.	Sample collection was performed following use of chewing gum, and thorough mouth-rinsing with water for an unspecified period of time.
n/a	Current study	11.4 ± 4.5	n/a	^1^H NMR analysis.	As described herein.

## Data Availability

The data presented in this study are available in this article.

## Supplementary Materials

The following supporting information can be downloaded at: https://www.mdpi.com/article/10.3390/metabo13070792/s1, Figure S1: Plots of STN ratio against NH_4_^+^ ion concentration; Figure S2: Comparison of the standard calibration curve and standard addition methods; Table S1: Standard calibration curve-determined NH_4_^+^ ion concentrations (mmol./L) in *n* = 27 WMSS samples collected during this study.
