# Supplementary material for: Detection and Quantification of Ammonia as the Ammonium Cation in Human Saliva by 1H NMR: A Promising Probe for Health Status Monitoring, with Special Reference to Cancer
Source: Metabolites. 2023 Jun 26;13(7):792. doi: 10.3390/metabo13070792 (PMC10383521; doi:10.3390/metabo13070792)
Supplement: Supplementary file 1 [file metabolites-13-00792-s001.zip › metabolites-2474978-supplementary.pdf]

Supplementary Materials

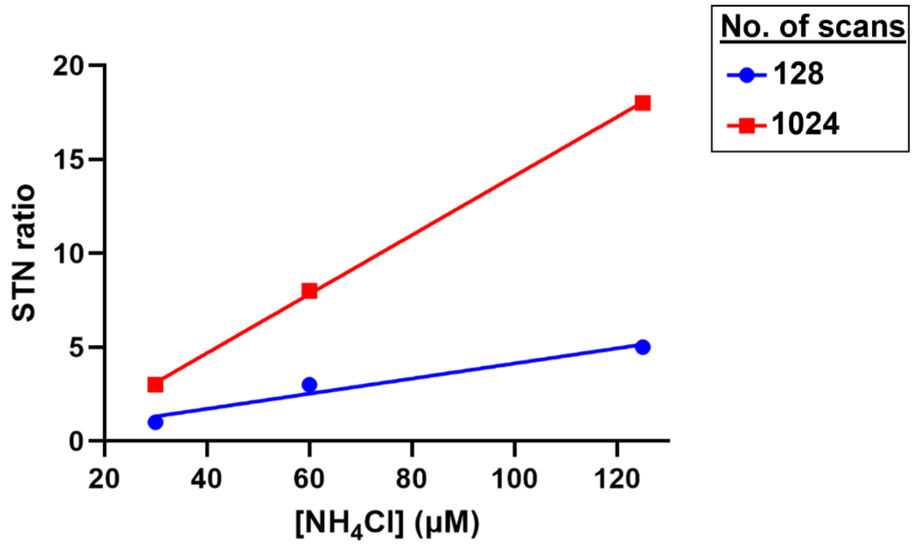

Figure S1. Plots of STN ratio against NH_4^+ ion concentration. These plots show dependence of the STN ratio on low levels of NH_4^+ ion concentration (added as ammonium chloride, NH_4Cl) using either 128 (blue) or 1,024 (red) ^{1}H NMR scans at an operating frequency of 600 MHz (µM = µmol./L). Spectra were acquired according to the description in Section 2.3 using the ROBUST-5 pulse sequence.

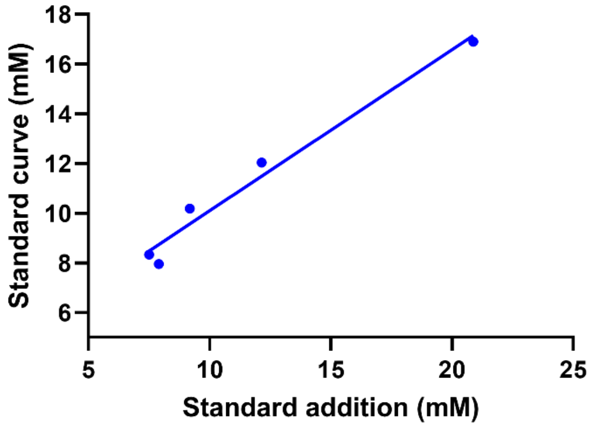

Figure S2. Comparison of the standard calibration curve and standard addition methods. Plot of WMSS NH_4^+ concentrations (mM = mmol./L) estimated from the standard calibration curve and standard addition method (SAM) bioanalytical strategies. This plot displays a strong linear relationship between the two different methods deployed for the analysis of n = 5 WMSS evaluation samples.

Sample Code	Salivary [NH_4^+] (mmol./L)
1	16.90
2	6.76
3	8.33
4	6.00
5	7.96
6	2.99
7	14.79
8	14.15
9	11.66
10	18.52
11	15.22
12	13.93
13	14.17
14	15.89
15	17.62
16	4.48
17	13.44
18	13.39
19	14.19
20	10.18
21	11.56
22	10.63
23	6.50
24	16.57
25	12.04
26	6.74
27	3.01

Table S1. Standard calibration curve-determined NH_4^+ ion concentrations (mmol./L) in n = 27 WMSS samples collected during this study.
